# Chicago Dental Society

**Published:** 1886-03

**Authors:** A. W. Hoyt


### PROCEEDINGS.

### Proceedings of the Chicago Dental Society.

At the February meeting of the Chicago Dental Society, a motion was introduced instructing the Secretary of the Society to furnish the dental journals with a copy of a resolution passed at the January meeting, endorsing the action of what is known as the Buffalo Conference of dentists. Before the motion was passed, it was suggested and understood that the reasons of those who opposed its passage and declined to vote one way or the other on the subject, should be published with the resolution. In obedience to these instructions, you will find below the resolution and the approved minutes of the Society in reference to this subject.

Dr. A. J. Swasey offered the following resolution, which was duly seconded: "Resolved, that this Society endorse the action of the Conference at Buffalo as regards the International Medical Congress." This called out an animated discussion in which Drs. Ottofy, Brophy, Crouse, Allport, and Baldwin participated. It was claimed that the Congress was going to be a failure, and if a failure, the Dental Section would of necessity also be a failure.

The resolution was opposed on the ground that as a society we had no right to take any such action as was contemplated in the resolution. The International Medical Congress, it was said, was in no sense a delegate body, and delegates as such would not be received by the Congress. It was open to any and all persons holding the degree of M. D., in regular medicine and such other gentlemen as should be invited to membership by the Executive Committee. Consequently, membership in the Congress was an individual and private matter, with which societies had nothing whatever to do. Neither this nor any other society had a right to handicap its members or any one else by passing such a resolution. Those who were trying to organize an International Dental Congress had a perfect right to do so, provided they felt that their duty lay in that direction; and those engaged in the work of the section would interpose no objection. The attempt to break up the section, that a Dental Congress might be made a success, was unjust and unmanly. Work in the section had been commenced long before the Buffalo meeting and much that was preliminary had already been done, and the attempt to stop it was the heighth of presumption and assurance, the right to do which was emphatically denied. The resolution is simply another attempt to manufacture public opinion against the section on Dental and Oral Surgery in the Congress, and to make it odious to have anything to do with it.

The very men in Chicago, who were at first exceedingly anxious for the section and flooded the country with letters and circulars, urging its organization, when the section was organized and they discovered that it could not be manipulated to their interest and that of their friends, went deliberately to work, (and that too within a week) to destroy it. This was months before the trouble between the factions among the medical men had been developed, and which was inaugurated at New Orleans. That it is on account of the trouble in the medical profession that the instigators of the opposition oppose this section, is a pretense, a subterfuge. They dare not state their real object, which was to get control of the section if they could; but if they could not, to organize an International Dental Congress.

Those who spoke in favor of the resolution, claimed that this Society was competent to act in the premises, and had a right to place itself on record as deeming the organization of the section inexpedient under the present circumstances.

Furthermore, the Congress was a delegate body, and medical societies had a right to send delegates and they would be received as such.*

The Congress, it was claimed, should be open to all dentists,

---

*The rule which governs the membership of the Congress reads as follows :

INTERNATIONAL CONGRESS, RULE FIRST.

"The Congress shall consist of members of the regular profession of medicine, who shall have subscribed their names on the and that unless it was so arranged, all had better stay away. At this juncture, Dr. Baldwin moved that the resolution be laid upon the table for one month, as the matter was an important one, and the members should have an opportunity to consider it before casting their votes. This was duly seconded. The Chairman put the motion and it was carried.

Dr. Crouse objected to the ruling of the chair, and called for a standing vote. The vote was again taken and declared lost by a small majority. A vote on the resolution was then called for and carried, the opposition previously declaring that for reasons before mentioned, they would not vote, and it was a matter with which societies had no right to meddle, that it was a matter between the Congress and those eligible to membership, and who wished to attend. A. W. Hoyt, D. D. S.

Recording Secretary.

